# PDMS Micropatterns Coated with PDA and RGD Induce a Regulatory Macrophage-like Phenotype

**DOI:** 10.3390/mi14030673

**Published:** 2023-03-17

**Authors:** Hoang Lan Pham, Da Hyun Yang, Woo Ri Chae, Jong Hyeok Jung, Thi Xoan Hoang, Nae Yoon Lee, Jae Young Kim

**Affiliations:** 1 Department of Life Science, Gachon University, Seongnam 13120, Gyeonggi-Do, Republic of Korea; 2 Department of BioNano Technology, Gachon University, Seongnam 13120, Gyeonggi-Do, Republic of Korea

**Keywords:** regulatory macrophages, micropatterns, polydopamine, arginylglycylaspartic acid, THP-1

## Abstract

Regulatory macrophages (Mreg) are a special cell type that present a potential therapeutic strategy for various inflammatory diseases. In vitro, Mreg generation mainly takes 7–10 days of treatment with chemicals, including cytokines. In the present study, we established a new approach for Mreg generation using a three-dimensional (3D) micropatterned polydimethylsiloxane (PDMS) surface coated with a natural biopolymer adhesive polydopamine (PDA) and the common cell adhesion peptide motif arginylglycylaspartic acid (RGD). The 3D PDMS surfaces were fabricated by photolithography and soft lithography techniques and were subsequently coated with an RGD+PDA mixture to form a surface that facilitates cell adhesion. Human monocytes (THP-1 cells) were cultured on different types of 2D or 3D micropatterns for four days, and the cell morphology, elongation, and Mreg marker expression were assessed using microscopic and flow cytometric analyses. The cells grown on the PDA+RGD-coated 3D micropatterns (20-µm width/20-µm space) exhibited the most elongated morphology and strongest expression levels of Mreg markers, such as CD163, CD206, CD209, CD274, MER-TK, TREM2, and DHRS9. The present study demonstrated that PDA+RGD-coated 3D PDMS micropatterns successfully induced Mreg-like cells from THP-1 cells within four days without the use of cytokines, suggesting a time- and cost-effective method to generate Mreg-like cells in vitro.

## 1. Introduction

Macrophages are innate immune cells that play important roles in the immune response to infectious agents and tissue injuries. Compared to other immune cells, the properties and functions of macrophages are dependent on their tissue microenvironment [1]. Macrophages undergo phenotypic alterations in response to tissue-specific signals, such as soluble factors, including growth factors and cytokines, or to those derived from direct interactions with environmental cells in tissues [1,2,3]. Macrophages are mainly classified into two subtypes, which can be derived from monocytes or resting macrophages in response to different stimuli, such as inflammatory or wound healing signals: pro-inflammatory (M1) and anti-inflammatory (M2) phenotypes [4,5]. In addition to these subtypes, regulatory macrophages (Mreg) have recently drawn attention because of their ability to suppress inflammatory and T-cell responses [6,7]. Thus, Mreg can be considered as a potential therapeutic strategy to suppress inflammation, which is a serious problem in various inflammatory diseases and organ transplantation. Conventionally, Mreg are generated from monocytes in vitro by stimulation with chemicals, including cytokines, and are maintained under consecutive induction and differentiation culture conditions for 7–10 days [8]. These chemical-induced induction and differentiation processes for Mreg would take a long time and a lot of money to obtain the final products [9]. Direct interactions between monocytes and components of the extracellular matrix (ECM) are crucial for macrophage differentiation [10]; however, physical factors can be used as an alternative approach to induce macrophage differentiation [11]. Indeed, several physical factors, such as substrate stiffness, roughness, and micropatterned topography, are known to regulate the phenotypic and functional plasticity of macrophages [11,12,13,14,15]. Different levels of substrate stiffness can control THP-1 cell differentiation into M1 or M2 macrophages [16]. Two-dimensional (2D) micropatterns with 20 μm-wide lines of fibronectin promote cell shape elongation of macrophages and facilitate M2 macrophage differentiation [11]. Surface roughness also affects the differentiation of macrophages; rough substrates induce M1 macrophage polarization, whereas smooth substrates promote M2 macrophage differentiation [13].

Polydimethylsiloxane (PDMS) is a polymer that is widely used for the fabrication of microfluidic chips and cell culture dishes [17]. However, the PDMS surface has limitations with regards to cell culture because it provides lower cellular adhesiveness [18]. To combat this issue, polydopamine (PDA) can be used to coat PDMS surfaces to stabilize cell interactions and control the adhesion of different cell types [19]. Consistently, we previously demonstrated that PDMS surfaces coated with PDA are suitable for cell adhesion and proliferation [20]. Nevertheless, PDA is not capable of communicating with cells in a manner akin to that of the ECM [21]. ECM molecules interact with integrins on the cell surface, which can initiate intracellular signaling that regulates diverse cellular processes, such as cell proliferation, cell death, differentiation, and migration [22]. Among integrins, arginylglycylaspartic acid (RGD) was recently used as a potential linker to facilitate the interaction of cells and biomaterials [23,24]. As an integrin-binding ligand, the RGD motif was also reported to mediate the macrophage attachment [25]. Hence, RGD can attract cells to interact with co-coated material PDA, which can attenuate the oxidative stress and the damage on the mitochondria of cells [26].

In the present study, we used RGD, which is the most common peptide motif responsible for cell adhesion and interaction with the cell adhesion protein integrin [27], to generate a PDA+RGD-coated PDMS surface as an alternative approach to mimic cell–cell or cell–ECM interactions. The aim of the present study was to examine whether Mreg-like phenotypes can be induced by culturing human monocyte THP-1 cells on fabricated PDMS micropatterns coated with PDA+RGD, which contain grooves of different widths, in the absence of any known chemical inducers of Mreg.

## 2. Materials and Methods

### 2.1. Reagents

Phorbol-12-myristate 13-acetate (PMA) was purchased from Cayman Chemical (Ann Arbor, MI, USA). Dulbecco’s phosphate-buffered saline (DPBS), RPMI-1640 culture medium, and fetal bovine serum (FBS) were purchased from Welgene, Inc. (Daegu, Republic of Korea). Dopamine hydrochloride, RGD, and dexamethasone (Dex) were purchased from Sigma-Aldrich (St. Louis, MO, USA). SU-8 3005 and SU-8 developer were purchased from MicroChem (Round Rock, TX, USA). PDMS prepolymer (Sylgard 184) and a curing agent were purchased from Dow Corning (Midland, MI, USA). Norland Optical Adhesive (NOA) 63 was purchased from Norland Products (Jamesburg, NJ, USA). Tris-HCl was purchased from Biosesang (Seongnam, Republic of Korea). Ethylenediaminetetraacetic acid (EDTA) was purchased from Amresco Inc. (Solon, OH, USA). Granulocyte-macrophage colony-stimulating factor (GM-CSF) and interferon (IFN)-γ were purchased from R&D Systems (Minneapolis, MN, USA). Pluronic F-127 solution and antibiotic–antimycotic solution were purchased from Invitrogen Corp. (Waltham, MA, USA). 1,25-dihydroxy vitamin D3 (VitD3) was purchased from Toronto Research Chemicals Inc. (Toronto, ON, Canada). 

### 2.2. Fabrication of 2D Micropattern

**Figure 1:**
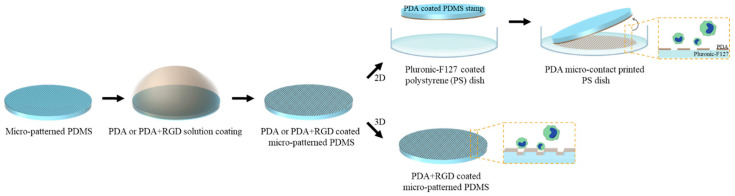
Fabrication process of polydopamine (PDA) micro-contact printed 2D patterns and PDA+ arginylglycylaspartic acid (RGD) coated 3D micropatterned polydimethylsiloxane (PDMS) for cell culturing.

A linearly aligned micropattern was fabricated using photolithography and soft lithography techniques. First, an SU-8 3005 photoresist was spin-coated on a silicon wafer at 1000 rpm for 35 s, followed by soft baking at 95 °C for 5 min. The SU-8 coated wafer was then exposed to UV light (365 nm, 15 mW·cm^−2^) for 14 s using a mask aligner and post-baked on a hot plate at 95 °C for 3 min. After immersion in the SU-8 developer solution and shaking at 70 rpm for 3 min, the wafer was thoroughly washed with isopropyl alcohol and dried at 65 °C for 1 min. A mixture of PDMS prepolymer and a curing agent at a ratio of 10:1 (*w/w*) was degassed and poured onto the SU-8 master mold and cured at 80 °C for 90 min. After the cured PDMS replica was carefully separated from the master mold, it was used for soft lithography. NOA 63, a UV-curable polymer, was poured onto the replicated PDMS and covered with an O_2_ plasma-treated PET film. The PDMS-NOA 63-PET film assembly was then exposed to UV light (365 nm) for 2 min before the PDMS was peeled off to produce the complementary micropatterns in the NOA 63 mold. The NOA 63-PET film mold was then fixed to a petri dish with the micropatterned side facing up, and the PDMS mixture of the prepolymer and a curing agent prepared at a ratio of 5:1 (*w/w*) was poured and cured under the same conditions described above. PDMS was used as a stamp to form a 2D micropattern using the microcontact printing technique. The PDMS stamp was coated with the PDA solution for 6 h at room temperature. To prepare the PDA solution, dopamine hydrochloride powder was dissolved to 2 mg·mL^−1^ in 10 mM Tris-HCl solution. The 6-well plate was treated with 0.2% Pluronic F-127 solution for 1 h, washed thrice with DPBS, and air-dried. The PDA-coated stamp was sterilized with 70% ethanol and washed with DPBS (Figure 1). After thorough drying, the micro-patterned side of the PDMS stamp came into direct contact with the Pluronic F-127 coated 6-well plate, and a 37 g weight was placed on the mold for 10 min.

### 2.3. Fabrication of 3D Micropattern

An NOA63-PET film mold was used to fabricate the 3D micropatterned PDMS substrates. The PDMS prepolymer and curing agent were mixed at a ratio of 10:1 (*w/w*), and the mixture was poured onto the NOA63-PET film mold and thermally cured. The micropatterned PDMS substrates engraved with an average depth of 8–10 µm were coated with the PDA solution or the PDA mixed with RGD (PDA+RGD) solution prior to use as cell culture substrates (Figure 1). The PDA solution was prepared in the same manner and concentration as mentioned above, and the PDA+RGD solution was obtained by dissolving RGD powder to a concentration of 0.04 mg·mL^−1^ in the PDA solution. The PDMS substrates were immersed in the PDA or PDA+RGD solution and coated for 6 h at room temperature before being thoroughly washed with deionized water, followed by ethanol and UV treatment in the hood for cell culture.

### 2.4. X-Ray Photoelectron Spectroscopy (XPS)

X-ray photoelectron spectroscopy (XPS) was performed to characterize the chemical compositions of the different PDMS surfaces. The measurements were performed using an AxisHsi (Kratos Analytical, Stretford, UK) equipped with a mono-gun aluminum X-ray radiation source (1486.6 eV) and a pass energy of 40 eV. The pressure in the chamber was below 5 × 10^−9^ Torr, and the take-off angle was set at 45°. The anode voltage was 13 kV, and the current was 18 mA. The binding energy of C1s (284.6 eV) was used as the reference.

### 2.5. Cell Culture and Treatment

The human monocytic cell line THP-1 (Korean Cell Line Bank, Seoul, Republic of Korea) was cultured in an RPMI-1640 medium supplemented with 10% heat-inactivated FBS and 1% antibiotic–antimycotic solution. The cells were maintained at 37 °C in a 5% CO_2_ humidified incubator. The resting macrophages were polarized from human monocytic THP-1 cells by treatment with 10 ng/mL PMA for two days. On day 3, the cells were washed with DPBS to remove non-adherent cells, followed by the detachment method described previously by Chen et al. [28], which included the use of 5 mM EDTA to detach the adherent cells, recovery by DPBS supplemented with 2.5% heat-inactivated FBS, and, finally, seeding onto micropatterns. After 2 days, the cells on the micropatterns were harvested using 5 mM EDTA and recovered by DPBS supplemented with 2.5% heat-inactivated FBS for further characterization. All the detachment steps were performed on ice.

### 2.6. Flow Cytometry

To determine the expression of the cell surface proteins, 1 × 10^6^ cells were seeded in each culture dish (the samples were repeated three times in each group). After the differentiation period, the cells were incubated with primary antibodies for 30 min at 4 °C, washed thrice with DPBS, and then incubated for 30 min with PE-conjugated or FITC-conjugated secondary antibodies at 4 °C. After washing, the cells were re-suspended in 0.4 mL DPBS and analyzed using a Cytomics FC500 MLP (Beckman Coulter Inc., Fullerton, CA, USA). For measurement of the intracellular protein expression, the cells were fixed by incubation with 4% formaldehyde in DPBS for 10 min, followed by permeabilization with 0.1% Triton X-100 for 10 min at room temperature prior to staining with the primary antibodies, and then with PE-conjugated secondary antibodies. The following antibodies were used for flow cytometry: CD163 and CD206 from Santa Cruz Biotechnology (Dallas, TX, USA); CD209 from Serotec (Bio-Rad, Hercules, CA, USA); CD274 from eBioscience Inc. (San Diego, CA, USA); MER-TK from R&D Systems (Minneapolis, MN, USA); DHRS9 from Abnova (Taiwan, China); and TREM2 from Biolegend (San Diego, CA, USA).

### 2.7. Immunofluorescence

After treatment, the culture medium was removed, and the differentiated cells were fixed with 4% formaldehyde in DPBS, followed by permeabilization with 0.1% Triton X-100 for staining of the intracellular proteins. Subsequently, the cells were blocked with 2% bovine serum albumin in DPBS, incubated with primary antibodies against specific proteins, and then with PE-conjugated secondary antibodies. The images were obtained using a fluorescent microscope (CKX53; Olympus, Tokyo, Japan) and analyzed using ImageJ software (National Institutes of Health, Bethesda, MD, USA). 

The cellular elongation factor was measured according to the method described previously [11]. Briefly, the long axis (defined as the longest length of the cells) and short axis (defined as the length across the nucleus in a direction that is perpendicular to the long axis) were manually measured. The ratio of the long axis to the short axis was calculated as the cellular elongation factors. The calculation was repeated three times at three independent areas. 

For the fluorescent signal quantification, the fluorescent images were separated into RGB color channels. The green or red stacks were selected, and the threshold adjustment was applied to detect the fluorescent cells, which were subsequently analyzed for total areas, pixel counts, area fraction, average size, and integrated density. The relative fluorescent intensity corresponds to the integrated density. The quantification was repeated three times at three independent areas.

### 2.8. Statistical Analysis

The experiments were repeated at least three times, and all data are presented as the mean ± standard deviation (SD). Significant differences among groups were analyzed by one-way analysis of variance, followed by a post hoc test using SPSS version 12.0 (IBM, Armonk, NY, USA). The differences were considered statistically significant at *p* < 0.05.

## 3. Results

### 3.1. XPS Analysis

**Figure 2:**
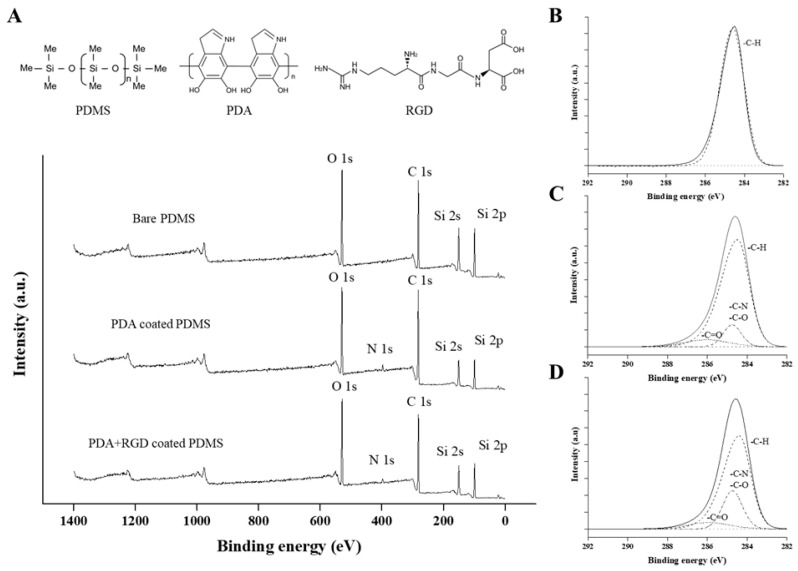
Schematic illustrations showing (**A**) X-ray photoelectron spectroscopy (XPS) survey spectrum results of the different PDMS surfaces; high-resolution spectra of C1s of (**B**) the bare PDMS, (**C**) the PDA-coated PDMS, and (**D**) the PDA+RGD-coated PDMS.

Figure 2 shows the results of the XPS analysis for the three types of PDMS surfaces. First, the survey spectrum of each PDMS surface is shown in Figure 2A. We could ascertain that the N1s peak was observed in both the PDA and PDA+RGD-coated PDMS survey spectra, whereas the Si peak was significantly decreased. These results are consistent with those of our previous studies on PDA- and RGD-coated PDMS [20,29]. This phenomenon could be explained by the reduction in the ratio of Si on the surface, as the PDMS was coated with PDA or PDA + RGD, and PDA is a catecholamine-based polymer that is comprised of nitrogen, while RGD is a peptide that is also comprised of nitrogen. The high-resolution spectra of C1s for bare PDMS are shown in Figure 2B. The bare PDMS spectra showed only the C–H bond peak, which was calibrated with reference to a binding energy of 284.6 eV, whereas the PDA- and PDA+RGD-coated PDMS had two additional peaks, as shown in Figure 2C,D. C–N and C–O bonds were detected at 286 eV, and the C=O bond was deconvoluted at 288.3 eV. Although there were no differences in the results of the C=O bond between the PDA and PDA+RGD-coated PDMS, we could confirm that the C–N and C–O bond intensities were greatly increased in the PDA+RGD-coated PDMS. Since PDA and RGD both contain ketones and aldehydes, as well as abundant amine groups, we can conclude that, when mixed, they could have provided more opportunities for C–N and C–O binding than when PDA was used alone. Overall, we confirmed that PDMS was successfully coated with the PDA and PDA+RGD solutions.

### 3.2. PDA-Microcontact Printed Micropatterns Induce Cell Elongation

**Figure 3:**
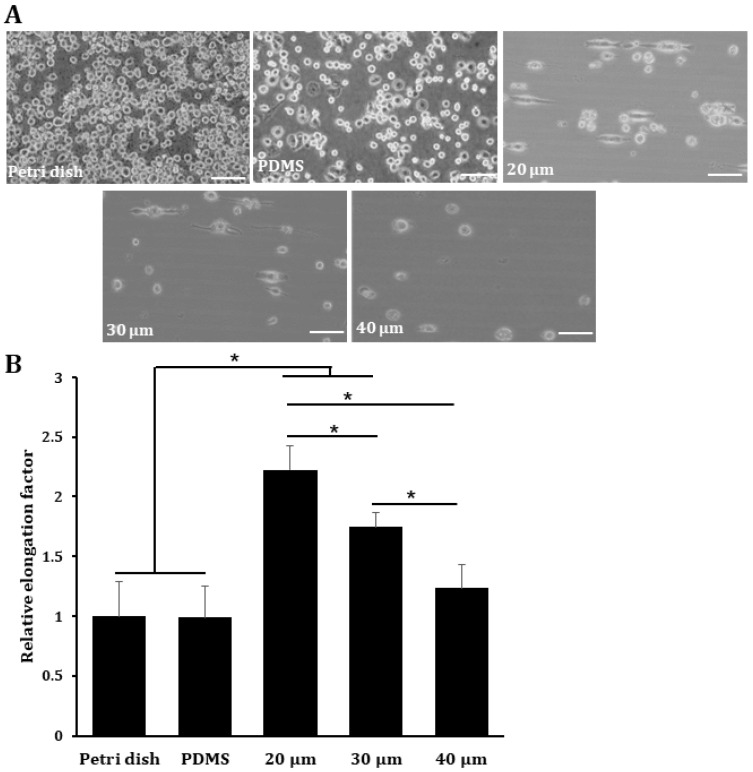
Cell elongation is induced when phorbol-12-myristate 13-acetate (PMA)-treated THP-1 cells are cultured on micropatterns printed with PDA. THP-1 cells were cultured on a petri dish, bare PDMS, or different-sized micropatterns in the presence of 10 ng/mL PMA for 4 days. (**A**) The cell morphology was analyzed by an inverted microscope at 100× magnification. The scale bar represents 100 μm. (**B**) Quantification of elongation factor of cells seeded on unpatterned and micropatterned surfaces. The images were analyzed by ImageJ to determine the elongation factor. The elongation factor was defined as the length of the longest axis divided by the length of the short axis across the cell nucleus, according to a previous study [11]. * *p* < 0.05.

Previous studies have demonstrated that Mreg/anti-inflammatory macrophages exhibit elongated morphology and cells express M2 phenotype markers even in the absence of exogenous stimuli [11,30]. Therefore, we first examined the cell morphology and the degree of cell elongation by microscopy. Bare PDMS or three different sizes of micropatterns printed with PDA were placed on a normal culture dish, and THP-1 cells were cultured on the plate in the presence of PMA for 4 days. The cells grown on the petri dish and bare PDMS surface exhibited round morphology, indicating that they did not adhere properly to the surface of the normal culture dish and bare PDMS, suggesting that these surfaces did not affect cell morphology (Figure 3A). However, cells grown on 20 µm-wide 2D micropatterns exhibited the most elongated morphology, followed by those grown on 30 µm-wide micropatterns, whereas the 40 µm-wide micropatterns did not promote cell elongation (Figure 3B).

**Figure 4:**
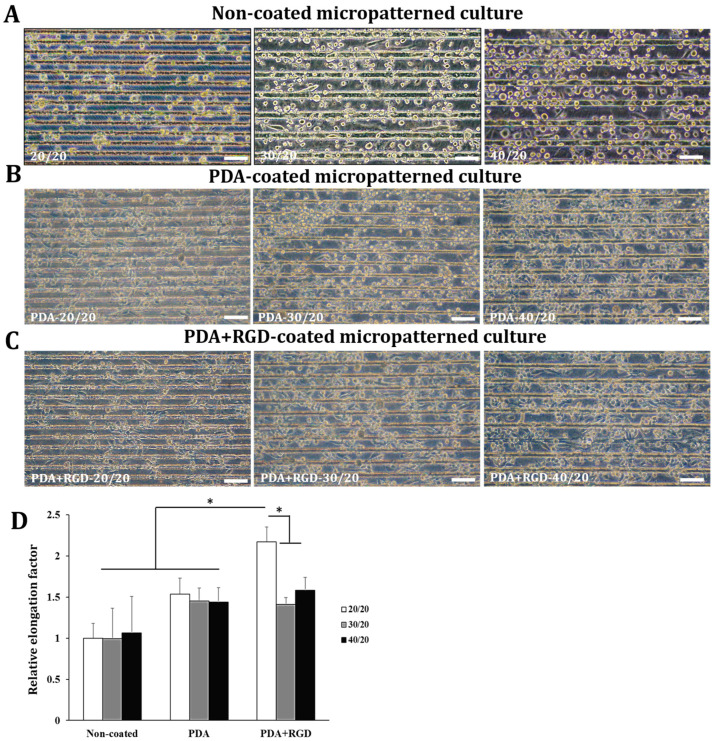
Cell elongation is most significantly induced when PMA-treated THP-1 cells are cultured on PDA+RGD-20/20 3D micropatterns. THP-1 cells were cultured on (**A**) a non-coated micropattern in the presence of 10 ng/mL PMA for 7 days or on (**B**) PDA-coated or (**C**) PDA+RGD-coated 3D micropatterns in the presence of 10 ng/mL PMA for 4 days. The cell morphology was analyzed by microscope at 100× magnification. The scale bar represents 100 μm. The images were analyzed by ImageJ to determine the elongation factor (**D**). * *p* < 0.05.

Next, we generated three types of PDMS micropatterns with different grooves, 20, 30, and 40 µm wide, spaced 20 µm apart between each groove (designated as 20/20, 30/20, and 40/20, respectively). To promote cell adhesion and elongation, RGD was added to the PDA solution and the micropatterned PDMS surface was coated with a PDA+RGD mixture (the images of the micropattern surfaces before cell seeding are shown in Figure S1). THP-1 cells were cultured on PDA-coated or PDA+RGD-coated 3D micropatterns with different widths for 4 days, while the cells were cultured on uncoated 3D micropatterns for a longer period (7 days) because of their insufficient adhesive capacity. Cell elongation was assessed by microscopic examination. As shown in Figure 4A–C, the cells grown on PDA- or PDA+RGD-coated 3D micropatterns exhibited significant changes in the cell morphology compared to those grown on non-coated 3D patterns. In particular, the cells grown on 20/20 PDA+RGD 3D micropatterns exhibited superior cell elongation compared to those grown on other micropatterns (Figure 4D). The elongation factor of the cells grown on PDA-coated 3D micropatterns were higher than those grown on non-coated micropatterns; the cells grown on 20/20 PDA-coated micropatterns exhibited a significant elongation factor (Figure 4D).

### 3.3. PDA+RGD-20/20 3D Micropatterns Strongly Enhance Expression of Mreg Markers

**Figure 5:**
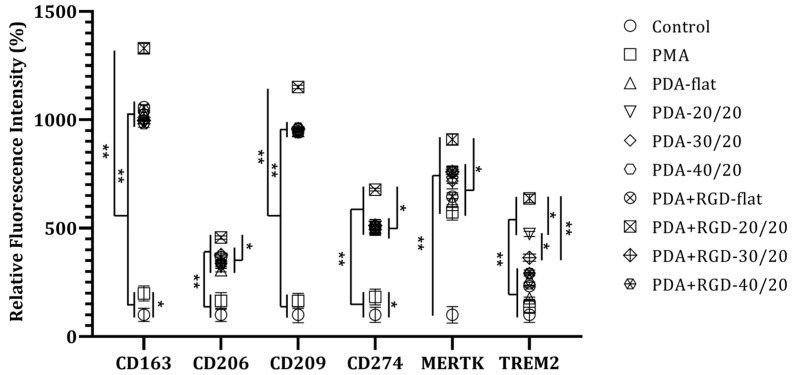
Regulatory macrophage (Mreg) markers expression is selectively enhanced by PDA+RGD-20/20 micropatterns. THP-1 cells were treated with PMA (10 ng/mL) only or treated with PMA for 2 days and then seeded on different types of micropatterns for 2 days. Relative levels of Mreg markers, such as CD163, CD206, CD209, CD274, and MER-TK and tumor-associated macrophage marker TREM2 were measured by flow cytometry. Control (o) designates DMSO-treated THP-1 cells. PDA-flat (∆) and PDA+RGD-flat (⊗) indicate PDA and PDA+RGD PDMS chips without micropatterns, respectively. * *p* < 0.05 and ** *p* < 0.005.

Next, we examined the expression of several Mreg markers, such as CD163, CD206, CD209, CD274, and MER-TK [31], and tumor-associated macrophage (TAM) marker TREM2, which exerts anti-inflammatory functions [32] by flow cytometry following culture of PMA-treated-THP-1 cells on different types of PDA- or PDA+RGD-coated 3D micropatterns. As shown in Figure 5, the cells grown on PDA+RGD-20/20 micropatterns exhibited the highest levels of all the examined Mreg markers and TAM marker TREM2. The cells grown on PDA-flat (PDA-coated but without microgrooves), PDA-coated 3D micropatterns, and 30/20 and 40/20 PDA+RGD micropatterns exhibited the second-highest levels of Mreg markers. On the other hand, the cells grown on PDA-20/20 micropatterns exhibited the second-highest levels of TREM2, suggesting that a 20 μm-width of the micropatterns is a more important factor for enhancing TREM2 expression than RGD coating (Figure 5). 

**Figure 6:**
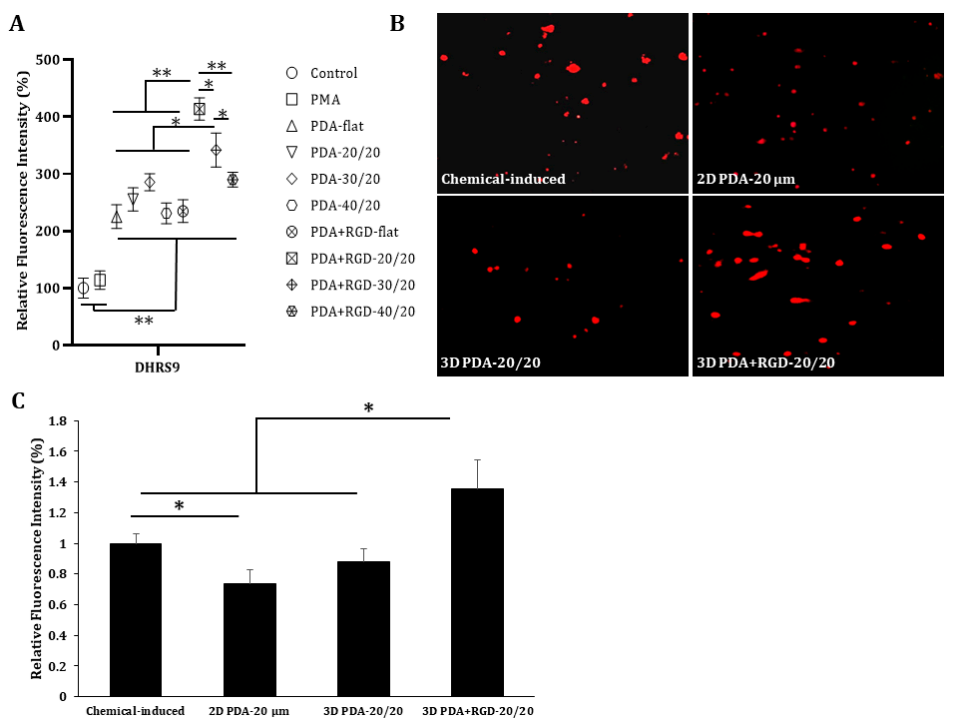
Expression of the Mreg stability marker is selectively enhanced by PDA+RGD-20/20 micropatterns. THP-1 cells were differentiated using different methods. In a chemical-induced protocol, THP-1 cells were treated with 10 ng/mL granulocyte-macrophage colony-stimulating factor for 2 days, followed by treatment with 10 ng/mL interferon-γ for 2 days. The cells were then treated with 10 nM dexamethasone for 1 day prior to treatment with 10 nM Vitamin D_3_ for 2 days [34]. In other groups, THP-1 cells were differentiated using 10 ng/mL PMA for 2 days and then seeded on micropatterns for 2 days. (**A**) The relative levels of DHRS9 were assessed by flow cytometry. (**B**) The levels of DHRS9 by different methods were compared by fluorescence microscopy. The images were taken at 200× magnification. (**C**) The fluorescence intensity was assessed by ImageJ. * *p* < 0.05 and ** *p* < 0.005.

Since dehydrogenase/reductase 9 (DHRS9) has been suggested as a marker for Mreg stability [33], we examined DHRS9 expression in PMA-treated cells grown on different types of PDA or PDA+RGD 3D micropatterns by flow cytometry. As shown in Figure 6A, cells grown on PDA+RGD-20/20 micropatterns exhibited the highest levels of DHRS9, whereas cells grown on PDA+RGD-30/20 micropatterns exhibited the second-highest levels of DHRS9. To compare the levels of this important Mreg stability marker between Mreg generated by our method and those generated by conventional methods, we generated Mreg using serial treatment with chemicals, such as granulocyte-macrophage colony-stimulating factor, interferon (IFN)-γ, dexamethasone, and vitamin D_3_ [34]. The levels of DHRS9 were assessed by fluorescence microscopy (Figure 6B,C). Consistent with the flow cytometric results, the cells grown on PDA+RGD-20/20 micropatterns exhibited the highest levels of DHRS9, which were higher than those induced by the conventional chemical methods (Figure 6C).

## 4. Discussion

In the present study, we demonstrated that human monocyte THP-1 cells cultured on fabricated PDMS micropatterns coated with PDA+RGD exhibited Mreg-like phenotypes even in the absence of biological stimulants, such as macrophage colony-stimulating factor (MCF-S), IFN-γ, or lipopolysaccharide, which are generally used to induce Mreg generation in vitro [8]. Mreg typically exhibits a fiber-like morphology and expresses cell surface markers, such as CD163, CD206, CD209, CD274, MerTK, and TREM2 [31] and the stability marker DHRS9 [33]. Consistently, in the present study, the cells grown on PDA+RGD-coated 3D micropatterns exhibited an elongated fiber-like morphology and expressed Mreg markers, suggesting an Mreg-like phenotype. 

Cells in isolation cannot regulate cellular processes, such as cell growth, differentiation, survival, and death. In particular, macrophages must continuously interact with ECM components to maintain specific phenotypes and functions [10]. Fibronectin plays a key role in cell adhesion and the functional activation of monocytes and macrophages [35,36,37]. The RGD sequence (Arg–Gly–Asp) is the most common motif found in fibronectin, and is the site of attachment via interaction with integrins on the cell surface [38]. Thus, fibronectin and its RGD motif have been used by several groups to promote cell differentiation [39], as well as to control cellular function [40]. We previously demonstrated that a PDA-coated PDMS culture surface enhances the adherent ability of human umbilical vein endothelial cells and bone marrow-derived mesenchymal stem cells [20]. To further promote cell adhesion and differentiation, RGD was used in addition to PDA, and the addition of RGD seemed to enhance cell adhesion on PDA-coated micropatterns, as demonstrated by cell elongation and expression of Mreg markers.

The interaction of cells with the extracellular environment through both soluble and physical factors can determine cell morphology. Changes in cell morphology can create biochemical signals and determine cell fate [41,42,43,44,45,46]. Mreg induced by conventional methods possess an elongated morphology [31] and cell elongation can act as an important factor for M2, but not M1 polarization [16,47,48]. McWhorter et al. used a micropatterning approach to obtain elongated macrophages exhibiting the M2 phenotype and found that cell elongation itself without exogenous cytokines leads to M2 polarization. They further demonstrated that this micropattern-induced M2 polarization is reversible when the cells are treated with inhibitors of actin and actin/myosin contractility, suggesting a micropattern-associated cytoskeleton function [11]. In the present study, PDA+RGD micropatterns with a 20 μm-width were found to be the best platform to induce cell elongation and expression of Mreg markers among the various widths tested. However, the reason underlying why a 20 μm-width 3D micropattern possesses the best capability to induce Mreg-like phenotype is unclear. One possible reason for this may be associated with the fact that the average cell size of macrophages is approximately 20 µm; therefore, the use of a 20/20-micropattern probably restricts cellular spread and, hence, directs cell elongation compared to wider sizes of micropatterns. 

Mreg are a potential therapeutic strategy for the treatment of various inflammatory diseases because of their potent suppressive effect on inflammatory and T-cell responses. Human Mreg are generally derived from peripheral blood monocytes in vitro and are stimulated with MCSF for 6 days following stimulation with IFN-γ, and require contact with a plastic surface and serum factors [33,49,50]. These methods require 7–10 days to induce the desired Mreg-like phenotype. In this respect, our 3D micropatterning approach with PDA+RGD has some advantages because with this approach, it only takes 4 days to induce Mreg-like cells with an elongated morphology and expression of Mreg markers without the use of relatively expensive cytokines.

However, our study has some limitations. We used the cell line THP-1 to generate Mreg-like cells and only phenotypically characterized Mreg-like cells. Another limitation is the lack of a functional test to verify the capacity of Mreg on Treg generation. Therefore, in future experiments, it is necessary to establish Mreg-like cells using primary monocytes (e.g., PBMCs or BMDMs) and further investigate the functional characteristics of these cells to ensure Mreg functions, such as anti-inflammation and regulatory T cell expansion capacities. In addition, the amount of generated Mreg is another issue. In several clinical research studies, the amount of required cells ranged from 0.5 to 10 × 10^6^ cells per kg body weight, while our research can only generate 1 × 10^6^ cells per micropattern surface [51]. Hence, our long-term target is improving the method to generate sufficient cells for clinical application. 

In conclusion, we demonstrated that a mixture of PDA and RGD can be successfully polymerized and coated on a micropatterned PDMS surface. Furthermore, the co-deposition of the two chemicals improved cell differentiation compared to coating with PDA alone. We successfully induced an Mreg-like phenotype in THP-1 cells within 4 days using PDA+RGD-coated 3D micropatterns in the absence of cytokines, suggesting a time- and cost-effective method to generate Mreg-like cells in vitro. The application of our PDA+RGD micropatterns could be extended to further studies on the differentiation of other macrophage phenotypes, as well as different cell types.

## 5. Conclusions

In this study, we coated PDA in combination with RGD on a PDMS surface to create a 3D culture platform for the generation of Mreg. To evaluate the potential 3D culture platform of a PDA+RGD-coated PDMS surface as a suitable surface for Mreg, human monocytic THP-1 cells were cultured. The efficacy of the micropattern as a differentiation factor was verified by the successful differentiation of THP-1 into Mreg, confirmed by protein markers expression. Furthermore, the cells seeded on 3D culture platforms were much more stable than 2D culture platforms, suggesting that both patterns and dimensional factor played an important role in the generation of Mreg. These results may contribute to the investigation of the effect of physical microenvironments on the generation of Mreg in vitro in a cost- and time-effective manner, suggesting a new approach to generate Mreg without using expensive materials.

## Supplementary Materials

The following supporting information can be downloaded at: https://www.mdpi.com/article/10.3390/mi14030673/s1, Figure S1: Microscopic images of the PDA+RGD-coated micropatterns before cell seeding. After the fabrication and characterization steps of the micropatterns, the surface patterns of PDA+RGD with different grooves, 20, 30, and 40 µm wide, spaced 20 µm apart, were captured by the inverted microscope at 100× magnification.
