# Supplementary figures and images for: PDMS Micropatterns Coated with PDA and RGD Induce a Regulatory Macrophage-like Phenotype
Source: Micromachines (Basel). 2023 Mar 17;14(3):673. doi: 10.3390/mi14030673 (PMC10052727; doi:10.3390/mi14030673)

**Figure S1. PDA+RGD-micropatterned surface before cell seeding**

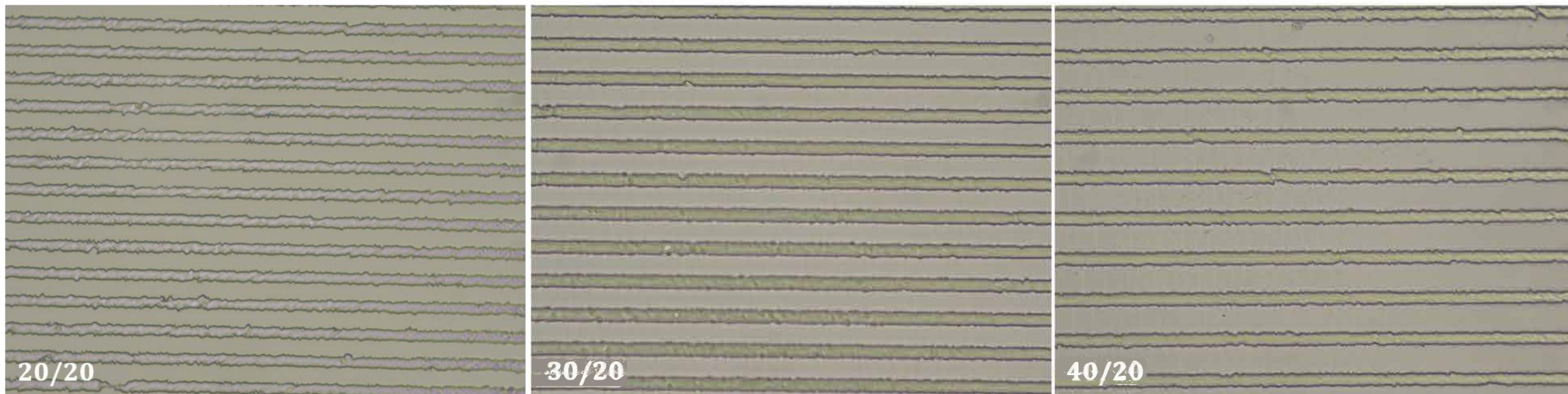

Supplement: Supplementary file 1 [file micromachines-14-00673-s001.zip › micromachines-2242728-supplementary.pdf]
